# Introducing a Simple Tool of Patient Self-Assessment of Wrist Range of Motion

**DOI:** 10.3390/life14080997

**Published:** 2024-08-10

**Authors:** Maximilian C. Stumpfe, Kaya Beneke, Raymund E. Horch, Andreas Arkudas, Wibke Müller-Seubert, Aijia Cai

**Affiliations:** Department of Plastic and Hand Surgery, Laboratory for Tissue Engineering and Regenerative Medicine, University Hospital Erlangen, Friedrich Alexander University Erlangen-Nürnberg FAU, Krankenhausstraße 12, 91054 Erlangen, Germany; kayabeneke@gmail.com (K.B.); wibke.mueller-seubert@uk-erlangen.de (W.M.-S.);

**Keywords:** telerehabilitation, range of motion assessment, hand rehabilitation, telemedicine, goniometer

## Abstract

Hand disorders can reduce wrist range of motion (ROM). The SARS-CoV-2 pandemic highlighted challenges in routine follow-up exams, making telemedicine a viable solution. This study evaluates the feasibility and accuracy of patient self-measured wrist ROM using a self-designed goniometer template. The template was designed to measure flexion/extension and radial/ulnar abduction movements. A cohort of 50 adults (25 males/25 females) participated in this prospective study. The exclusion criteria included wrist immobilization and ages outside of 18–65 years. Participants self-assessed their wrist ROM with the goniometer template. Measurements were independently performed by a student and a specialist using standard goniometry, as well as a resident using the self-designed goniometer. The results were blinded for unbiased analysis. Mean differences in ROM varied across movement directions, with minimal differences for ulnar abduction and more substantial deviations for radial abduction, extension and flexion. The patient–specialist comparison showed deviations below 5 degrees for flexion and ulnar abduction in 50% of cases. Telemedicine, expanded by the COVID-19 pandemic, offers significant potential for hand rehabilitation. Current methods of ROM assessment lack cost-effectiveness and simplicity. Our method, demonstrating comparable accuracy for most movements, provides a cost-effective, reliable alternative for remote ROM assessment, enhancing telemedicine practices in hand rehabilitation.

## 1. Introduction

The range of motion (ROM) is a key measurement of wrist function [1]. The current gold standard for quantifying ROM in clinical practice is the goniometer, which is a non-invasive, low-cost and easy-to-use tool [2,3]. Regular ROM assessments are crucial for monitoring therapeutic progress.

These are important in postoperative rehabilitation, traumatic hand injuries and chronic hand disorders [4]. The importance of ROM measurement in postoperative rehabilitation lies in the recording of rehabilitation progress. Following traumatic hand injuries such as distal radius fractures, regular re-evaluation of the range of motion is necessary to assess the restoration of function. Systematic and regular monitoring is also required for chronic conditions such as osteoarthritis of the wrist, carpectomy and hand pain due to various other pathologies [5,6]. 

Nevertheless, the implementation of routine follow-up examinations has faced challenges, particularly during the SARS-CoV-2 pandemic.

Various nations have implemented diverse strategies to mitigate the spread of SARS-CoV-2 infections [7]. Especially during periods of lockdowns, telemedicine has emerged as a promising solution to maintain patient–provider connections. Consequently, there has been a notable expansion and increased utilization of telemedicine platforms [8]. Studies showed that the rate of telemedicine consultations increased by around 12% after the SARS-CoV-2 pandemic [9]. In particular, younger patients, including the working population, seem to profit from this development as elderly patients perceive telemedicine as challenging and, therefore, prefer in-person consultations [10]. 

The use of telemedicine has significant potential in the field of hand rehabilitation [4]. Telerehabilitation offers solutions to many issues associated with traditional rehabilitation. Between 25% and 73% of patients report having missed direct follow-up appointments [11,12,13]. Various reasons are cited for this issue, including cost, lack of time and insufficient understanding of the importance of hand therapy aftercare. Additionally, the availability of specialized hand therapists in rural areas is low [4]. Telerehabilitation can help mitigate these issues to some extent.

Currently available systems for measuring hand movement in the context of telemedical consultations primarily include smartphone-based measurements and sensor-based systems [4]. However, these systems are often time-consuming, expensive and challenging for the patient to apply [4]. Therefore, we propose that our developed template offers a simple, quick, and cost-effective method for measuring wrist movement within the context of telemedicine and rehabilitation.

This study aims to analyze the self-assessment of wrist ROM by patients utilizing a novel goniometer template. The objective is to determine whether these self-measured values offer a reliable method of assessing ROM suitable for telemedicine applications compared to standard measurements performed by a specialist. By investigating the feasibility and accuracy of self-reported ROM measurements, this research endeavors to contribute to the optimization of telemedicine practices in the context of hand rehabilitation.

## 2. Materials and Methods

### 2.1. Participants

In this prospective study, a cohort of 50 adult participants was recruited. The volunteers consisted of either hospitalized patients (those undergoing hand surgery were excluded), hospital staff other than physicians (e.g., nursing staff) or friends/acquaintances of the authors. The selection process aimed to mirror the population distribution of Germany, ensuring the attainment of a representative sample. Participants aged 18–65 years were included to represent the working population, who would, in the authors’ opinion, predominantly make use of telemedicine applications. Specifically, the distribution was as follows: 2 individuals aged 18–20 years, 4 participants aged 21–24 years, 15 individuals aged 25–39 years, 23 contributors aged 40–59 years, and 6 participants aged 60-64 years. The exclusion criteria encompassed individuals currently undergoing wrist immobilization. Participants were first informed verbally about the study. Afterwards, they received written information about the study and its protocol. The volunteers received sufficient time to consider their participation. The participants were informed that their decision for or against contribution would have no effect on their future medical treatment.

The study was granted approval by the institutional research ethics committee (22-238-B) and is in accordance with the 1964 Helsinki Declaration and its later amendments or comparable ethical standards.

### 2.2. Design of Template

**Figure 1:**
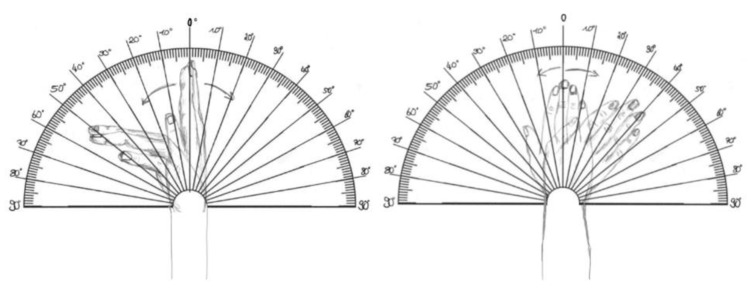
Self-designed goniometer template to determine the range of motion.

The self-designed goniometer template includes two semicircular protractors, each marked with degree measurements from 0° to 90° in both clockwise (for left hands) and counterclockwise (for right hands) directions. The size of the template is a DIN A3 (297 mm × 420 mm or 11.7 inches × 16.5 inches) sheet of paper to ensure positioning of the complete hand onto the template whilst still being able to identify the degree values for the patient. The 0° marking is drawn in the center. The grading is performed in increments of 10°. These lines are extended beyond the length of an average hand/finger so that it is possible to use them as an extension of the fingers during flexion and extension. The middle finger acts as a “pointer” during radial and ulnar abduction. A marked line on the protractor ensures that the transition between the wrist and forearm is correctly aligned. The motions have been illustrated in the background to make them easier to understand (see Figure 1).

In a preliminary study, residents and specialists of hand surgery (*n* = 13) performed measurements on the same wrist of one subject using the self-designed goniometer and a standard goniometer. To avoid bias, the measurements were taken at least 7 days apart. The measured wrist was not affected by pain or any circumstances that could alter the range of motion between the two measurements. The measurements demonstrated a difference of approximately 5° for each movement, indicating that clinical comparability with the gold standard can be anticipated [14]. Detailed results of the preliminary study are presented in the Results section.

### 2.3. Assessment

Participants were requested to provide demographic and clinical information, comprising age, sex, profession, hand dominance and any prior surgical interventions on the upper extremities. Additionally, they were instructed to complete the above-mentioned goniometer template (refer to Figure 1) to assess the ROM of their wrists. The participants were asked to place the sheet on a flat surface and position their forearm on the marked area, ensuring that the transition between the wrist and the forearm was aligned with the vertical line at 0° on the goniometer as shown on the template (see Figure 1). Depending on the movement, the hand was placed on the sheet either with the palmar surface (for radial and ulnar abduction) or with the ulnar lateral side. They were asked to move their wrist to its maximum range in both directions while keeping their fingers extended. These measurements were carried out in gravitationally unloaded conditions.

The final step was to record the corresponding range of motion (ROM) and enter it into the provided table. The tables (4-field panel) were primarily subdivided into extension/flexion and ulnar abduction/radial abduction. Radial abduction was described as towards the thumb and ulnar abduction as towards the little finger so that a layman was able to understand the movements. It was also divided into right and left.

Subsequently, wrist measurements were conducted three times. This involved two measurements performed by both a student and a specialist utilizing the gold-standard method of goniometry. In order to match the gravitationally unloaded conditions of the self-examination of the ROM via template, the measurements with the goniometer were also carried out with the hand positioned on a flat surface.

**Figure 2:**
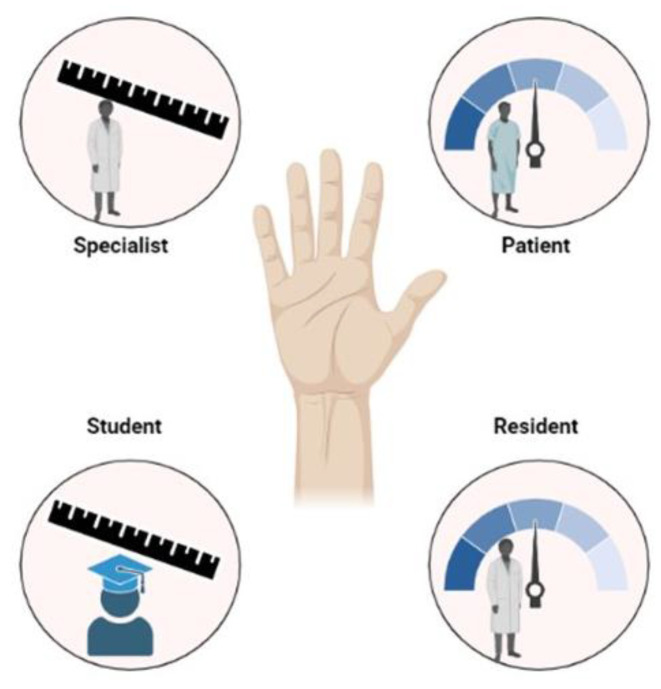
Overview of the assessment methods and the designated persons responsible for the measurements (created using BioRender.com, accessed on 28 June 2024).

Additionally, a resident utilized the goniometer described above for the third measurement (Figure 2).

The measurements were placed in sealed envelopes until the completion of patient recruitment. This protocol was implemented to maintain a double-blind study design, thereby preventing any potential biases during the analysis phase.

### 2.4. Statistical Analysis

Descriptive statistics were expressed as median, standard deviation, and range. A paired t-test was used for comparison of academic vs. non-academic patients. A *p* value < 0.05 was considered significant. The comparison of the measurements carried out by the student, resident, specialist, and the patients was analyzed via one-way ANOVA or Friedman test, as appropriate. We used Tukey’s multiple comparisons test as post hoc tests between all four measurements. Statistical analysis was conducted with GraphPad Prism 9.0 (GraphPad Software Inc., La Jolla, CA, USA).

We based our sample size on other studies on this topic. Of the 28 studies cited in the scoping review by Kuchtaruk et al., the number of patients included ranged from 5 to 171, with a mean sample size of 41, so we set a sample size of 50 [4].

## 3. Results

### 3.1. Pre-Study

The pre-study showed significant differences for two movements (flexion and ulnar abduction). The mean difference was 5.9° for flexion and 4.4° for ulnar abduction (Table 1).

### 3.2. Study

A total of 50 participants were enrolled in the study, comprising 25 males and 25 females. The majority of the cohort (*n* = 47, 94%) was right-handed. The participants were categorized into academics and non-academics according to their professions. An academic education was found in 38% (*n* = 19) and a non-academic education in 29 patients (58%). Six patients (students, pensioners) (4%) could not be categorized. Nine (18%) patients had a history of wrist treatment. These can be summarized as fracture of the ulna (*n* = 5), carpal tunnel surgery (*n* = 3) and tenovaginitis stenosans de Quervain (*n* = 1).

**Figure 3:**
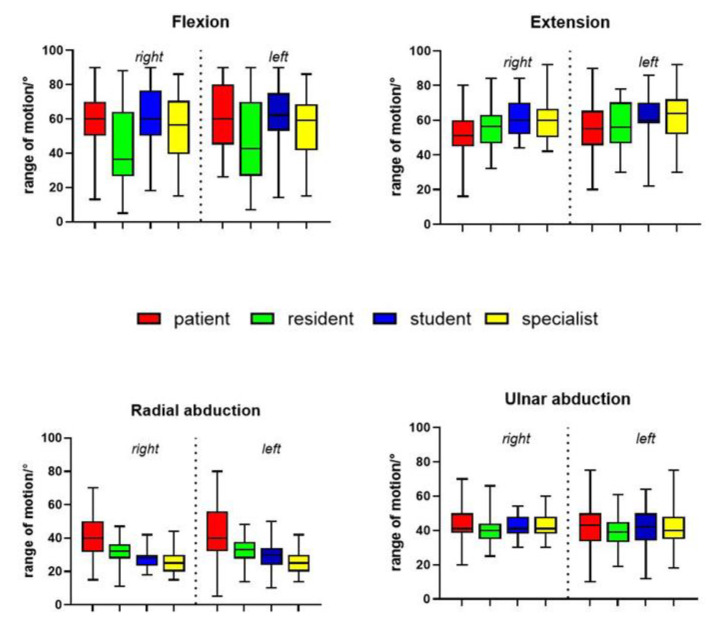
Graphical illustration of the ROM, divided into the individual examiners, movement and sides.

The statistical analysis revealed a spectrum of mean differences ranging from a minimum of 0.2 degrees to a maximum of 19.7 degrees across all movement directions. Specifically, the range of mean differences for each direction of movement were as follows: 0.2°–9.2° for extension, 0.9°–19.7° for flexion, 0.4°–18.0° for radial abduction and 0.2°–4.2° for ulnar abduction (Table 2; Figure 3).

Notably, there were no statistically significant differences in measured ulnar abduction. Conversely, significant disparities in range of motion (ROM) were observed for radial abduction, extension and flexion (Table 3).

### 3.3. Subgroup Analysis: Academics vs. Non-Academics

Of the 24 comparative examinations between patients and observers (student, resident, specialist), 71% (*n* = 17) showed no significant differences in the subgroup of academics (Table 4). The mean difference for the significant deviations was 11.6 degrees for radial abduction (10.4–13.5; *n* = 4), 9.9 degrees (9.3–10.5; *n* = 2) for extension and 15.3 degrees (*n* = 1) for flexion. In the non-academics, there were no significant differences in 16 (67%) comparative analyses. A mean deviation of the significant differences of 10.5 degrees (7.4–12.2; *n* = 3) was found for radial abduction, 8.7 degrees (8.5–8.8; *n* = 2) for extension and 13.5° (8.4–17.4; *n* = 3) for flexion.

The comparison of the subgroups of academics and non-academics showed that in 71% (*n* = 17), there was no significant difference between the measurements. In seven measurements, there was no difference in measured values between the examiners and one of the subgroups, while there was a measurable difference in the other subgroup.

The difference in mean deviations from the significant results to the examiners’ measurements was small between academics and non-academics: 7.1° for radial abduction (*n* = 3), 5.7° for extension (*n* = 2) and 5.2° for flexion (*n* = 2).

## 4. Discussion

The expansion of telehealth has seen significant acceleration, particularly due to the COVID-19 pandemic, which has catalyzed the integration of remote healthcare solutions into routine practice [4,15,16]. Between 25% and 73% of patients do not consistently perform their exercises at home or do not attend therapy appointments [11,12,13]. Reasons for this include lack of time and the inconvenience of attending face-to-face appointments [11,12,13]. In addition, access to trained hand therapists is often limited in rural areas [4]. These challenges can be optimized with telemedicine [17]. Nevertheless, remote ROM measurements taken by the patient themself must be accurate and reliable to ensure appropriate treatment planning.

Current available methods include smartphone photographs with manual measurements of joint angles or angle measurement applications with direct assessment of the ROM [18]. The disadvantage of these two smartphone-based options is a high amount of time required to evaluate the ROM directly or afterwards and the dependency on the patient to provide an accurate recording [19,20]. However, these disadvantages are not due to the patient’s lack of will, but are because of an increased difficulty in achieving the proper tilt angle for later evaluation of the photograph-based joint measurement. Determining the optimal patient positioning for telemedical assessment of the range of motion is often a problematic challenge [21]. This leads to incorrect documentation of the ROM, limiting the rehabilitation process [4].

Our self-designed goniometer template offers advantages in terms of handling and evaluation. Brief instructions make it easy for all patients to carry out the correct measurement and evaluation. The challenges of finding the correct tilt of a smartphone are not an issue. Patients simply have to perform the movement, measure the angle and document the corresponding ROM. It is a simple method that requires little effort. Regardless of an individual’s educational background, the method can be effectively applied.

In contrast to smartphone-based ROM examinations—which use the sensors and camera built into the smartphones—the sensor-based assessments use special devices, mostly wearable gloves, to record the range of motion.

Sensor-based remote ROM assessments are highly efficient for the patients and the healthcare provider [14]. The above-mentioned disadvantages of the smartphone-based methods are not present. The measurements show the highest inter/intraobserver reliability [22]. The major disadvantage of sensor-based telerehabilitation is the associated costs as the sensors are expensive to purchase [22]. This seems unaffordable if patients receive them for regular remote assessment. The method described by us provides a cost-effective procedure for the regular determination of ROM by the patient in the context of telemedicine. Our method is so intuitive to use that, unlike other methods, it does not require supervision or implementation by the author [1,23,24,25] and can be used independently.

The results of our study show a rate of 50% for deviations below 5 degrees of ROM for flexion and ulnar abduction of patients compared to specialists. In 25% of the cases, there is a deviation below 10 degrees (*n* = 2) and 25% of the mean values show a deviation of 15 degrees. This applies particularly to radial abduction. We can, therefore, conclude that for flexion and ulnar abduction, the presented method of ROM self-measurement is in accordance with the gold standard of the goniometer examination as the clinical difference is lower than the 5° obligatory for clinical significance [14]. We also consider the variance between 5° and 10° to be unproblematic. Studies on intra- and inter-rater reliability showed that the reliability can range between 7 and 9° when using a standard goniometer [26,27]. On the other hand, the clinical relevance of a deviation of this extent for the treatment decision must be discussed.

Radial abduction measurements revealed a higher deviation, suggesting the need for further refinement of the instructions. We assume that radial abduction might lead to a further radial abduction of the middle finger by flexion of the MCP joints, which might have led to an incorrect interpretation as the patient was asked to use their middle finger as a pointer for the scale. This might have led to the difference of 15° in the mean values provided by the patient or specialist since a specialist would use the dorsum of the hand as one of the axes of the angle. A comparison of the literature on alternative ROM assessments indicates that, in many cases, certain directions of movement are less accurate. For example, the study by Marti-Puente et al. showed that radial and ulnar abduction have low accuracy in the photographic determination of ROM [28]. Kassay et al. showed the same for ulnar abduction in photographically determined ROM measurements [1]. This is confirmed in a number of other studies [20,29,30,31,32]. Noteworthy in this context are the results of the pre-study. Here, radial abduction only showed a deviation of 0.1 degrees between the gold-standard goniometer and our template. It can, therefore, be assumed that the measurement by the patient was more problematic, which could possibly be resolved by adapting the instructions for use (e.g., video instructions).

All in all, the method described is a simple and cost-effective method for examining the ROM of the wrist. The comparison of academic and non-academic employees showed that the designed template is easy to use regardless of educational background. An adaptation for measuring the ROM of the finger joint is also possible [33,34].

The authors see the above-mentioned measurement as a useful tool to enable close monitoring of the patient’s treatment progress after the baseline examination in an unloaded condition by the surgeon and instruction of the patient in the use of the template. With the help of the template, it is possible to recognize stagnation of the ROM at an early stage and to adapt, and, if necessary, extend the postoperative exercise treatment. It also offers the examiner the opportunity to offer telemedical aftercare in order to reduce the time and effort for the patient in the event of long journeys to attend in-person appointments and to make a recommendation for further treatment. The introduction of our self-designed goniometer template can be seen as part of a broader trend towards the digitization and decentralization of healthcare. As telemedicine continues to evolve, integrating simple, reliable tools for self-assessment becomes increasingly important. The goniometer template is not only a solution for current telemedicine applications but also a step towards more patient-centered care models. By providing tools that patients can use independently, we promote a sense of ownership and engagement in their rehabilitation process. This empowerment can lead to better adherence to treatment plans and potentially improved outcomes. Furthermore, the goniometer template can serve as a valuable tool in educational settings, helping patients understand the importance of monitoring their ROM and recognizing the progress they are making.

In addition to its use in telemedicine, the goniometer template could be beneficial in clinical settings where quick assessments are needed without the availability of a specialist. For example, in primary care or general practice settings, where specialists may not be immediately available, the template can provide preliminary assessments that guide further referrals and treatments.

Another potential application of the goniometer template is in research settings. Researchers studying hand function and rehabilitation can utilize the template for large-scale studies where multiple measurements are needed across different sites. The simplicity and low cost of the template make it an ideal tool for such purposes, ensuring consistency and reliability in data collection.

A limitation of our study arises from a few deviations between the template measurement and the gold-standard goniometer. Future studies should incorporate multiple observers to validate these findings and enhance the measurement accuracy.

## 5. Conclusions

In conclusion, our goniometer template method provides a feasible and reliable solution for remote ROM assessment in hand rehabilitation, addressing key challenges associated with other telehealth technologies and offering a promising tool for enhancing patient care in telemedicine.

The integration of this goniometer template into telemedicine practices holds the potential to significantly improve the accessibility and quality of hand rehabilitation. By enabling patients to perform accurate measurements independently, we can enhance the continuity of care, reduce the need for in-person visits, and support the overall goals of patient-centered care and empowerment. Future research and refinement of the template will further solidify its role in the evolving landscape of telehealth and hand rehabilitation.

## Figures and Tables

**Table 1:** Results of the pre-study to validate the template; n.s. = not significant.

	Flexion	Extension	Radial Abduction	Ulnar Abduction
Goniometer	79.5 ± 6.5	67.9 ± 67.9	26.5 ± 6.4	45.2 ± 6.4
Template	73.6 ± 7.2	68.2 ± 7.8	26.6 ± 2.9	40.8 ± 4.8
Mean difference	5.9	0.3	0.1	4.4
	*p* = 0.04	n.s.	n.s.	*p* = 0.02

**Table 2:** Tabular representation of the average range of motion in degrees and the corresponding standard deviation. Results are also broken down for the individual examiners and movement.

	Flexion		Extension		Radial Abduction		Ulnar Abduction	
	Right	Left	Right	Left	Right	Left	Right	Left
Patient	60.0 +/− 19.4	60.0 +/− 18.9	51.0 +/− 12.7	55.0 +/− 15.5	40.0 +/− 13.1	40.0 +/− 15.7	41.0 +/− 9.5	43.0 +/− 12.6
Resident	36.6 +/− 21.5	42.5 +/− 24.4	56.5 +/− 11.3	56.0 +/− 13.5	32.0 +/− 6.8	33.0 +/− 7.6	40.0 +/− 8.7	39.0 +/− 9.0
Student	60.0 +/− 19.4	62.0 +/− 17.0	60.0 +/− 10.1	60.0 +/− 11.2	28.0 +/− 5.2	30.0 +/− 7.7	41.0 +/− 6.6	42.0 +/− 10.2
Specialist	56.5 +/− 20.2	59.0 +/− 17.8	60.0 +/− 11.5	64.0 +/− 13.6	25.0 +/− 6.1	25.0 +/− 7.0	41.0 +/− 6.6	40.0 +/− 10.4

**Table 3:** Tabular presentation of the significant deviations between the investigators with the *p* value and the mean deviation; * = significant; ** = very significant; *** = extremely significant.

		*p*-Value	Mean Deviation
Radial abduction			
right—patient vs. student	***	<0.0001	13.2
right—patient vs. resident	***	<0.0001	9.2
right—patient vs. specialist	***	<0.0001	15.1
right—student vs. resident	**	0.0084	−4.1
right—resident vs. specialist	***	<0.0001	6.0
left—patient vs. student	***	<0.0001	13.5
left—patient vs. resident	***	<0.0001	11.1
left—patient vs. specialist	***	<0.0001	16.5
left—resident vs. specialist	**	0.0018	5.4
Extension			
right—patient vs. student	***	<0.0001	−8.5
right—patient vs. specialist	*	0.012	−7.4
right—student vs. resident	***	0.0002	6.5
Flexion			
right—patient vs. resident	***	<0.0001	15.3
right—student vs. resident	***	<0.0001	16.9
right—resident vs. specialist	**	0.0016	−10.9
left—patient vs. resident	***	0.0009	12.8
left—student vs. resident	***	<0.0001	14.7
left—student vs. specialist	**	0.005	7.2

**Table 4:** Detailed description of the subgroup analysis with presentation of the individual movements, side, examiner comparison, *p*-value and mean deviation; A = academic education; NA = non-academic education; n.s. = not significant; * = significant; ** = very significant; *** = extremely significant.

			*p*-Value		Mean Deviation	
Radial abduction						
	A	NA	A	NA	A	NA
right—patient vs. student	**	***	0.0099	0.0005	10.4	11.9
right—patient vs. resident	n.s.	**	0.0594	0.0037	8.3	7.4
right—patient vs. specialist	**	***	0.0077	0.0002	13.5	12.2
left—patient vs. student	*	n.s.	0.0190	0.8231	11.0	1.7
left—patient vs. resident	n.s.	n.s.	0.1494	0.4695	5.9	3.4
left—patient vs. specialist	**	n.s.	0.0088	0.9994	11.5	0.3
Ulnar abduction						
right—patient vs. student	n.s.	n.s.	0.8500	0.1714	1.7	4.2
right—patient vs. resident	n.s.	n.s.	0.8015	0.0625	1.9	5.0
right—patient vs. specialist	n.s.	n.s.	0.9459	0.7625	1.3	1.9
left—patient vs. student	n.s.	n.s.	0.9949	0.8231	0.6	1.7
left—patient vs. resident	n.s.	n.s.	0.3837	0.4695	4.2	3.4
left—patient vs. specialist	n.s.	n.s.	0.9974	0.9994	0.6	0.3
Extension						
right—patient vs. student	**	***	0.0011	0.0005	9.3	8.8
right—patient vs. resident	n.s.	n.s.	0.9990	0.9939	0.3	0.6
right—patient vs. specialist	n.s.	*	0.7297	0.0362	3.7	8.5
left—patient vs. student	**	n.s.	0.0046	0.3907	10.5	3.9
left—patient vs. resident	n.s.	n.s.	0.2339	0.9992	5.8	0.4
left—patient vs. specialist	n.s.	n.s.	0.1095	0.0982	8.3	6.4
Flexion						
right—patient vs. student	n.s.	n.s.	0.8962	0.9955	3.3	0.7
right—patient vs. resident	*	***	0.0176	<0.0001	15.3	17.4
right—patient vs. specialist	n.s.	n.s.	0.9501	0.0831	2.3	7.8
left—patient vs. student	n.s.	n.s.	0.2236	0.9996	6.2	0.3
left—patient vs. resident	n.s.	***	0.0750	0.0004	9.5	14.6
left—patient vs. specialist	n.s.	**	0.7566	0.0076	2.9	8.4

## Data Availability

The original contributions presented in this study are included in the article; further inquiries can be directed to the corresponding author/s.
